# Monitoring respiratory mechanics by oscillometry in COVID-19 patients receiving non-invasive respiratory support

**DOI:** 10.1371/journal.pone.0265202

**Published:** 2022-03-21

**Authors:** Chiara Torregiani, Chiara Veneroni, Paola Confalonieri, Gloria Maria Citton, Francesco Salton, Mohamad Jaber, Marco Confalonieri, Raffaele Lorenzo Dellaca’

**Affiliations:** 1 Department of Pulmonology, Azienda Sanitaria Universitaria Giuliano Isontina, Trieste, Italy; 2 Department of Electronics, Information and Biomedical Engineering (DEIB), TechRes Lab, Politecnico di Milano University, Milan, Italy; Ospedale Sant’Antonio, ITALY

## Abstract

**Background:**

Non-invasive ventilation (NIV) has been increasingly used in COVID-19 patients. The limited physiological monitoring and the unavailability of respiratory mechanic measures, usually obtainable during invasive ventilation, is a limitation of NIV for ARDS and COVID-19 patients management.

**Objectives:**

This pilot study was aimed to evaluate the feasibility of non-invasively monitoring respiratory mechanics by oscillometry in COVID-19 patients with moderate-severe acute respiratory distress syndrome (ARDS) receiving NIV.

**Method:**

15 COVID-19 patients affected by moderate-severe ARDS at the RICU (Respiratory Intensive Care Unit) of the University hospital of Cattinara, Trieste, Italy were recruited. Patients underwent oscillometry tests during short periods of spontaneous breathing between NIV sessions.

**Results:**

Oscillometry proved to be feasible, reproducible and well-tolerated by patients. At admission, 8 of the 15 patients showed oscillometry parameters within the normal range which further slightly improved before discharge. At discharge, four patients had still abnormal respiratory mechanics, not exclusively linked to pre-existing respiratory comorbidities. Lung mechanics parameters were not correlated with oxygenation.

**Conclusions:**

Our results suggest that lung mechanics provide complementary information for improving patients phenotyping and personalisation of treatments during NIV in COVID 19 patients, especially in the presence of respiratory comorbidities where deterioration of lung mechanics may be less coupled with changes in oxygenation and more difficult to identify. Oscillometry may provide a valuable tool for monitoring lung mechanics in COVID 19 patients receiving NIV.

## Introduction

Severe and critical respiratory failure characterised pandemic disease COVID-19 in about one-fifth of the cases [1] and stressed healthcare resources worldwide [2, 3]. In this context, non-invasive ventilation (NIV) modalities in severely de novo hypoxemic patients have spread inside and outside Intensive Care Units (ICU) [3, 4] to become first-line support. NIV reduced endotracheal intubations necessity and improved clinical outcomes [3, 5]. In pandemic COVID-19, NIV failure and the risks of delayed intubation have been debated [6, 7]. A recent review about COVID-19 non-invasive treatments underscored the lack of solid clinical predictors for non-invasive supports failure [8]. The decision to intubate can be based on an excessive work of breathing judged clinically [9], but often the intubation is a merely subjective decision of the physician in charge [6]. NIV failure is reported in case of decreased level of consciousness, exhaustion, refractory hypoxemia [10, 11], sepsis and hemodynamic instability [10] and it is related to clinical parameters of excessive work of breathing [6, 7]. In spontaneously breathing patients, objective measurement of respiratory effort, work of breathing and lung mechanics requires esophageal manometry. However, esophageal manometry is invasive, needs expertise, and produces discomfort for the patient with acute distress [6, 9], resulting not feasible in daily clinical practice.

The Forced Oscillation Technique (FOT), also known as Oscillometry [12], provides an effective, simple, and non-invasive approach for monitoring lung mechanics during spontaneous breathing at the bedside. A pressure oscillation is applied at the mouth, and the resulting flow is measured. The relationship between oscillatory pressure and flow provides the respiratory system’s resistance (Rrs) and reactance (Xrs) at the oscillation frequency. Depending on the oscillatory frequencies, different physiologic information can be obtained. Rrs tends to be largely frequency-independent in healthy adults in the range of 4–50 Hz and is mostly determined by the properties of the central aiways [12]. An increased Rrs at lower frequencies (increased Rrs frequency dependence) indicates ventilation heterogeneity [12]. Rrs frequency dependence can be quantified by the differences between Rrs at two different frequencies, for example, Rrs at 5 Hz minus Rrs at 19 Hz. Xrs is determined by both respiratory system inertance (related to the pressure needed to accelerate the gas column) and compliance. In adults, Xrs at 5 Hz (X5) is commonly considered sensitve to changes in the lung periphery [12]. Reduced X_5_ are associated with lower respiratory system compliance, higher peripheral airway resistance or lower lung volume[13, 14]. Within breath changes in X_5_ is sensitive to dynamic airway compression and increased difference between inspiratory and expiratory X_5_ (ΔX_5_) identify the presence of tidal expiratory flow limitation [15].

Due to the poor predictability of COVID-19 patients clinical evolution and the risk of operators contamination during patients’ examination we decided to apply FOT to patients treated with NIV beginning during the first COVID 19 wave in the spring of 2020. This pilot study reports these data and the feasibility of monitoring longitudinal changes of respiratory mechanics by FOT in COVID-19 patients with ARDS at the NIV treatment was applied. We evaluated patients’ tolerance to the measurements, the reproducibility of the results, the changes in lung mechanics with time and their relationship with the clinical characteristic of the patients to probe the presence of valuable clinical information.

## Materials and methods

We conducted an observational retrospective pilot study. The local Ethical Review Board approved the study (Comitato Etico Unico Regionale Friuli Venezia Giulia, CEUR ID #3306) and waived the informed consent due to the retrospective nature of the study. All the data collected by the clinician who followed the patients were anonymised before data analysis as per ethical review board requirement. The data were stored in a secure data system exchange provided by the regional IT provider Insiel.

### Study subjects

The study was conducted at the RICU (Respiratory Intensive Care Unit) dedicated to COVID-19 patients of the University Hospital of Cattinara, Trieste, Italy. FOT measurements were performed between April and May 2020 and then after October 2020 until December 2020, when the COVID unit was open and the FOT device available. Inclusion criteria were: i) laboratory-confirmed Sars-Cov-2 infection by RT-PCR test from a nasopharyngeal swab, ii) moderate-severe COVID-19 ARDS requiring non-invasive ventilation because of suboptimal saturation (SpO2 < 94%) with high flow oxygen support, and iii) Glasgow Coma Scale (GCS) of 15. Exclusion criteria were: i) sedative treatments, ii) anamnesis of cognitive impairment, iii) haemodynamic instability, and iv) age < 18-years. Participant selection followed a convenience series based on the timely availability of the research team.

### Patients’ management

At hospital admission, all the patients started treatment with prolonged low-dose methylprednisolone [16]. Within the first 24 hours in RICU, arterial blood gas was measured during HFNC support and the arterial oxygen partial pressure to fractional inspired oxygen ratio (PaO_2_/FiO_2_) was calculated. The same day, the blood chemistry data lactate dehydrogenase (LDH), D-dimer, lymphocyte count and creatinine were collected. Computed tomographic pulmonary angiography (CTPA) was requested according to clinical judgment during hospitalisation to elucidate pulmonary embolism suspect. Patients were treated with NIV using oronasal masks and NIV was maintained if tolerated and improved PaO_2_/FiO_2_ with reduction of respiratory rate. Daily NIV with cyclic prone positioning was alternated to increasing periods of HFNC until successful weaning.

### FOT measurements

Respiratory mechanics were measured by oscillometry in a seated position using a multifrequency signal comprising 5, 11, and 19 Hz (Resmon ProFULL, Restech Srl, Milan, Italy) during continuous electrocardiographic and arterial oxygen saturation monitoring. Ten spontaneous breaths were recorded, and the measurements were performed at least in triplicate and following the current guidelines [12]. HFNC was immediately restored after a measurement and kept between the repeated measurements for 3–5 min. Measurements were repeated at least twice for each patient. Oscillatory measures were performed on patients starting when periods of HFNC could be safely introduced between NIV sessions and the clinician considered secure the temporary HFNC removal. Tests were performed at least once a week and, when possible, twice a week with the last one taken at RICU discharge.

### Data and statistical analysis

All the clinical and laboratory data were captured from the electronic medical record. When available, computed tomographic pulmonary angiography during RICU hospitalisation or subsequent perfusion scintigraphy data were also recorded. Patients with less than two FOT measurements or with the last FOT measured taken more than 24h before discharge from the unit were excluded. Resistance and reactance at 5 Hz (R_5_ and X_5_, respectively) were computed as an average of the three repeated measurements, discarding one outlier, if needed, to provide R_5_ coefficient of variation (R_5__CV)<10%, as per current guidelines [12]. Z-score of R_5_ and X_5_ were obtained considering their predicted normal ranges [17]. The difference between resistance at 5 Hz and 19 Hz (R_5_-R_19_) was computed to evaluate the frequency dependence of the resistance that is related to the heterogeneity of ventilation. Inspiratory and expiratory R_5_ and X_5_ were also calculated. The difference between inspiratory and expiratory X_5_ (ΔX_5_) was computed to detect tidal expiratory flow limitation (EFL_T_) [13]. Data were corrected for the impedance of the viral and bacterial filter used. A linear mixed-model was used to test changes in R_5_ and X_5_ with time, considering fixed effects for the intercept and time plus random effect for the intercept for each patient. Correlations between oscillometry parameters and laboratory data, initial PaO_2_/FiO_2_ values were tested by Spearman test. Data were analysed using Matlab R2020b (MathWorks, Natick, MA, USA), SigmaPlot v11 (Systat Software, Inc., San Jose, CA, USA), and R version 4.0.4 (R Foundation for Statistical Computing, Austria).

## Results

A convenience series of 15 patients affected by COVID-19 critical pneumonia satisfying Berlin criteria for moderate-severe ARDS were enrolled. Ten patients were hospitalised between 08/04/2020 and 15/05/2020 during the first wave of COVID 19 and 5 patients at the beginning of the second wave between 28/10/2020 and 14/12/2020. Hospitalisation, anthropometric, clinical, laboratory and radiologic characteristics of the included patients are shown in Table 1. Clinical conditions that could impact respiratory function were the following. Three patients had a diagnosis of pulmonary embolism during hospitalisation (#2, #6, #10). One patient presented a previous diagnosis of respiratory comorbidity: patient #1 was affected by chronic obstructive pulmonary disease (COPD, 1). Another patient (#4) did not report pre-existing respiratory morbidities but had bronchospasms identified by auscultation during the first days of hospitalisation in RICU with no sign of heart failure and inhalation therapy was introduced. Only patient #1 was a smoker. Patients #8 and #9 were ex-smokers and quit smoking 40 and 20 years ago, respectively. All patients improved and were discharged to non-intensive medical wards.

**Table 1:** Patients data.

Patient	Age (y)	BMI (Kg/m^2^)	Comorbidities	Smoking	Days at hospital [at RICU]	CTPA proven pulmonary embolism	PaO_2_/FiO_2_ (mmHg) [FiO_2_%]	CRP (mg/L)	D-dimer (mg/L)	LDH (units/L)	Lymphocytes (counts/uL)	Creatinine (mg/dL)
**#1**	68	28	HT, COPD, pAF	Yes	55 [7]	-	146 [50]	132	0.38	117	420	0.71
**#2**	67	28	DM, HT	No	37 [31]	Yes	180 [85]	37	0.55	327	620	0.67
**#3**	79	28	rD, DM, HT	No	35 [16]	No	164 [71]	48	2.24	372	1750	1.19
**#4**	53	33	HT	No	18 [8]	No	192 [92]	175	0.81	346	500	0.77
**#5**	44	34	HT	No	29 [6]	-	170 [60]	61	0.20	385	670	0.73
**#6**	82	25	PAC, HT	No	55 [12]	Yes	167 [75]	75	4.43	432	900	0.83
**#7**	74	30	HT	No	9 [4]	No*	162[60]	328	0.9	401	690	0.84
**#8**	76	30	HT, pAF	Former	41 [19]	No	120[90]	66	1.6	403	450	1.09
**#9**	69	30	-	Former	44 [5]	No	172[100]	82.4	1.4	545	700	0.55
**#10**	74	26	BPH	No	19 [10]	Yes	170[80]	328	9.8	593	890	0.59
**#11**	43	30	DM	No	21 [5]	-	115[100]	36	0.36	341	1060	0.63
**#12**	64	30	DM	No	20 [8]	No	141 [70]	84	0.47	471	440	0.84
**#13**	62	20	-	No	17 [8]	No	62 [90]	86	0.93	236	560	0.89
**#14**	55	31	-	No	13 [5]	No	70 [60]	13	1.03	291	460	0.94
**#15**	67	29	HT	No	12 [8]	No	111[80]	132	0.88	343	420	0.92
**Median (IQR)**	67 (58;74)	30 (28;30)	1 BPH	1 Smoker 2 Ex-smokers	21 (17;39) [8 (5;11)]	3 Yes	162 (117;170) [80 (65;90)]	82 (54;132)	0.9 (0.5;1.5)	372 (334;417)	620 (455;795)	0.83 (0.69;0.95)
			1 COPD									
			2 pAF									
			1 rD			9 No						
			1 PAC			3 NA						
			4 DM									
			9 HT									

BMI = Body Mass Index; COPD = Chronic Obstructive Pulmonary Disease; pAF = permanent atrial fibrillation; HT = hypertension; DM = diabetes mellitus; rD = recent diverticulitis; PAC = prostatic adenocarcinoma; BPH = benign prostatic hyperplasia; Former smoker: stop at least 20 years ago; RICU = Respiratory Intensive Care Unit; CTPA = computed tomographic pulmonary angiography; PaO_2_ = arterial oxygen partial pressure; FiO_2_ = fractional inspired oxygen; CRP = C-reactive-protein; D-dimer. LDH = lactate dehydrogenate. All the laboratory data and and PaO_2_/FiO_2_ were sampled at first days at RICU. Our laboratory normal values are CRP <5mg/L; D-dimer <0.5 mg/L; LDH < 250; Lymphocytes between 1000-4000/uL and Creatinine between 0.5–1.3 mg/dL. PaO_2_/FiO_2_ was measured during HFNC support.

* patient 7 underwent negative pulmonary scintigraphy 31 days after discharge. Data are presented as discrete values (yes/no/former) or median with interquartile range for continuous values.

A single FOT measurement took less than 1 minute to be performed. All patients well-tolerated the measurements, possibly showing modest and transient reduction of oxygen saturation (SpO_2_) to 88–90% at the end of the tidal breathing with immediate restoration of the SpO_2_ with nose clips removal and restart of HFNC oxygen delivery. No discomfort was reported by the attending physician. Triplicate measurements presented an R_5__CV<10% in 55% of the cases. R_5__CV becomes <10% in the remaining cases after the exclusion of an outlier. Respiratory rate (22±9 breath/min at first measurement), tidal volume (0.88±0.44 L at first measurement), and minute ventilation (17±6 L/min at first measurement) during the measurements did not change in time.

**Fig 1:**
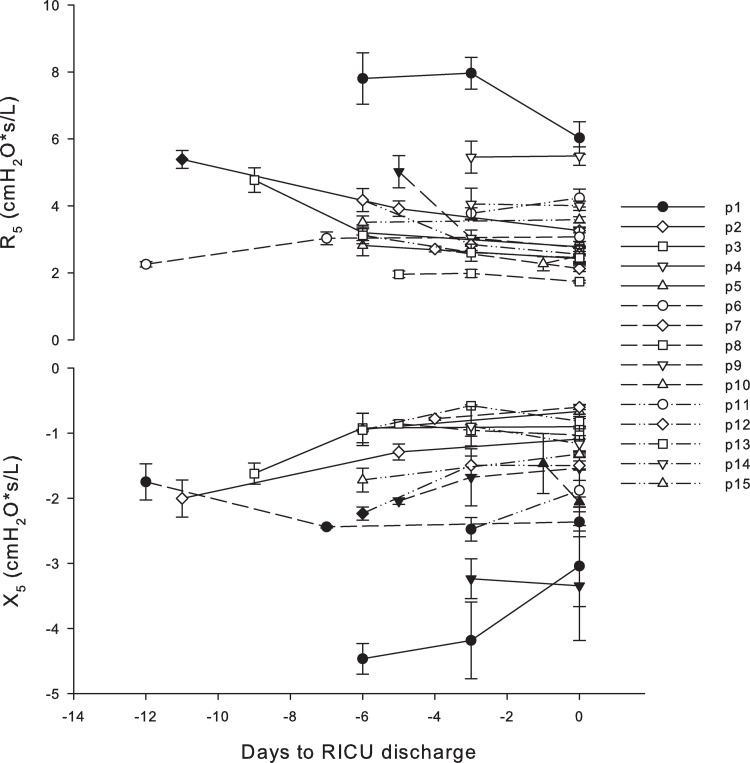
R_5_ and X_5_ for all the patients vs days prior to RICU discharge. Closed symbols identify altered R_5_ and X_5_ values compared to normal reference (Zscore > 1.645).

**Fig 2:**
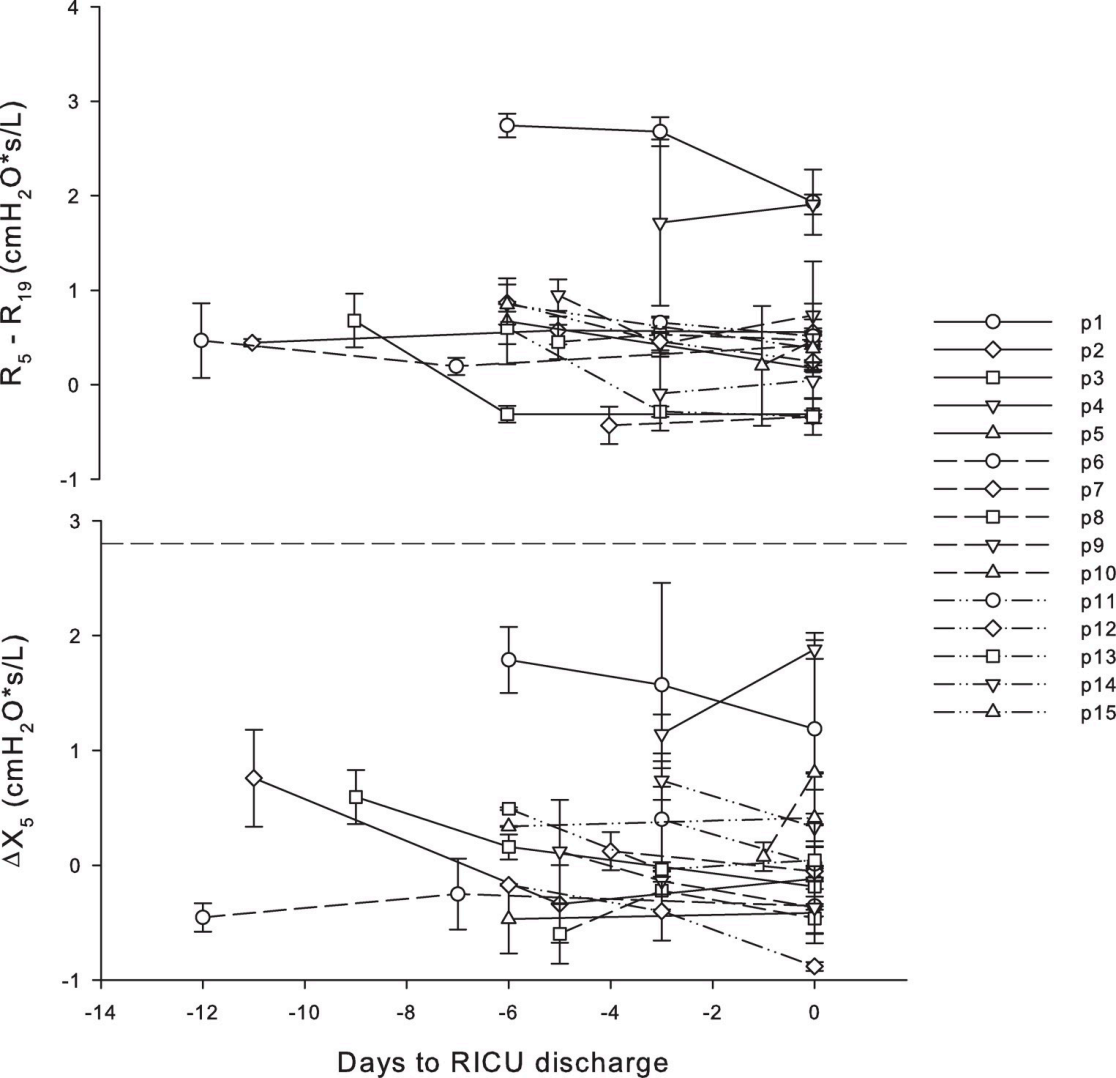
R_5_—R_19_ and ΔX_5_ for all the patients vs days prior to RICU discharge. Dashed line identifies the threshold for fully developed EFL_T_. No reference values are available in literature for R_5_-R_19_.

Time intervals between measurement sessions were 3.7±1.6 (mean ± standard deviation) days. On average, R_5_ significantly decreased (p<0.001) and X_5_ increased (p<0.001) during hospitalisation (Fig 1), with only two patients (#6 and #10) showing a worsening of oscillometry data. Our patients presented different lung mechanical conditions. At the first measurement, lung mechanics were abnormal in 8 out of 15 subjects. In particular, three patients presented increased R_5,_ five patients presented reduced X_5_ and two patients presented both increased R_5_ and reduced X_5_ (Table 2). R_5_ improved in all of them while X_5_ worsen in two of them. Four patients had still abnormal oscillometry data at discharge: one patient presented increased R_5_, three patients presented reduced X_5_ and one patient presented both increased R_5_ and reduced X_5_ (Table 2). Two patients (#1 and #4) had higher R_5_-R_19_ and mean ΔX_5_ (Fig 2) than the other patients. At the first measurement and discharge, the oscillometric parameters did not correlate with initial PaO_2_/FiO_2_ and laboratory data.

**Table 2:** Z-score of oscillometry measurements.

	First measurement		Last measurement	
Patient	R_5_ z-score	X_5_ z-score	R_5_ z-score	X_5_ z-score
**#1**	**3.06**	**4.95**	**2.09**	**2.68**
**#2**	**1.94**	1.52	0.06	-0.48
**#3**	0.44	-0.51	-1.59	-2.16
**#4**	0.90	**2.76**	0.92	**2.91**
**#5**	-1.53	-1.13	-2.06	-1.90
**#6**	0.42	**2.29**	1.52	**3.67**
**#7**	0.08	-0.47	-0.79	-0.99
**#8**	-1.14	-0.42	-1.56	0.04
**#9**	**2.62**	**2.80**	0.48	1.61
**#10**	0.57	**2.07**	0.92	**3.48**
**#11**	0.39	**2.97**	0.79	1.64
**#12**	0.96	**2.38**	-0.79	0.68
**#13**	**1.75**	0.88	0.75	0.20
**#14**	**1.69**	0.61	**1.66**	0.84
**#15**	0.66	1.43	0.74	0.46
**Median (IQR)**	0.66 (0.40;1.72)	1.52 (0.09;2.57)	0.74 (-0.78;0.92)	0.68 (-0.22;2.16)

Z-scores > 1.645 indicate increased R_5_ or decreased X_5_ values compared to normal reference values as reported by [17].

## Discussion

This pilot study aimed to test FOT feasibility in acute hypoxemic COVID 19 ARDS patients treated with non-invasive supports. To the best of our knowledge, this is the first report on the application of FOT to spontaneously breathing patients with COVID-19 acute respiratory failure. It was possible to perform FOT measurements in triplicate in all sessions for all patients and each test lasted less than one minute. Measurements were well tolerated and reproducible during the entire course of hospitalization.

Early reports about health personnel infected by COVID-19 [1] have addressed the risk of transmission during aerosol-generating procedures as a considerable burden and recommendations have been produced [18]. Coughing and heavy breathing increase the velocity and volume of air forced over the respiratory mucosa producing large amounts of respiratory particles [19]. Viral load during coughing atomises respiratory secretions more than tracheal intubation [20], exposing the healthcare personnel to the risk of viral droplets during physical examination. The possibility of applying FOT in this contest is of interest as it allows to monitor lung function in COVID 19 patients reducing the risk of viral droplets exposure. In fact, FOT only requires quiet breathing through a bacterial filter.

In our convenience sample, approximately half of the patients had normal R_5_ and X_5_ while at RICU, showing that hypoxemia in COVID-19 is not only determined by airway obstruction or lung volume de-recruitment. Normal X_5_ values are in line with previous studies reporting normal compliance in a minority of COVID 19 and non-COVID 19 ARDS patients [21–24]. X_5_ and PaO_2_/FiO_2_ were not correlated in our patients and this is in line with previous studies reporting no correlations between oxygenation and lung compliance in intubated or non invasively ventilated ARDS COVID 19 patients [23–25], but contrary to the data of Vandendunder et al. [26]. The lack of correlation between these variables also supports that in several patients oxygen requirement is mainly determined by other factors than reduced lung compliance and peripheral obstriction, such as, for example, ventilation perfusion mismatch or other factors related to pulmonary circulation. Recent literature reviews are clearing up the central role of thrombotic microangiopathy in the early phenotype of COVID 19 pneumonia that worsens the ventilation-perfusion ratio without especially weighting on lung compliance [27, 28].

FOT provides a quantitative and objective evaluation of lung mechanics that can be easily repeated at different time points, allowing monitoring changes in lung conditions. Therefore, the time course of the disease and the effects of treatments on lung mechanics can be evaluated. FOT data improved with time in most patients, even when the first measurement was within the normal range, showing a pattern more compatible with recovery from interstitial or very mild oedema into peripheral lung regions than those typical of extensive alveolar flooding [14, 29]. In our dataset, no patients failed NIV and the X_5_ improvement we found is in line with the improvement in lung compliance reported in AHRF patients with NIV success [21].

Four patients with different underlying conditions had still abnormal oscillometry data at discharge. One patient was affected by chronic obstructive pulmonary disease (COPD, #1). Another one (#4), despite not reporting pre-existing respiratory morbidities, had bronchospasms as identified by auscultation. In these two patients, oscillatory mechanics were consistent with the presence of an obstructive pattern shown by increased R_5_ values. They also showed the highest R_5_-R_19_ (the range of normality for this parameter is not reported in the literature) and ΔX_5_ values. ΔX_5_ values, despite not reaching the threshold for fully developed EFL_T_, suggests that some airways developed choke points during expiration. This quantitative information provided by FOT data can help tailor bronchodilator therapy and follow the clinical course of obstructed ventilated COVID-19 while reducing the biological hazard of physical examination.

The other two patients with abnormal FOT data at discharge (patient #6 and #10)did not show signs of obstruction nor EFL_T_ (normal R_5_ and ΔX_5_ close to 0), but were the only patients whose X_5_ worsened with time while at the RICU. These patients showed the worst initial D-dimer and evidence of pulmonary embolism at CTPA during hospitalisation in RICU. In these two patients, the abnormal oscillometry data may also be related to the pulmonary embolism that can lead to constriction of airways adjacent to the embolised lung segment and/or pulmonary edema, as previously reported [30]. If X_5_ worsening can predict acute or chronic worsening of clinical conditions remains to be evaluated. This could be valuable information since COVID 19 severe pneumonia needing invasive ventilation is characterized by a reduction in aerated lung surface with a progressive reduction in the compliance of the respiratory system [23, 24, 26], greater increase in the lung weight mainly due consolidated lung regions, and non-perfused areas that become predominant compared to COVID 19 severe pneumonia manageable with non-invasive support [31]. The majority of our patients had normal/near normal reactance, their gas exchange improved with time and they were discharged to medical ward. If oscillatory lung mechanics worsens in patients with less favorable progression leading to the need of mechanical ventilation requires future studies. Also, since about one-third of severe respiratory COVID 19 undergo long-lasting fibrotic-like changes in the lung [32] it will be interesting to evaluate if X_5_ worsening could predict fibrotic-like deterioration of lung parenchyma.

Our data suggest that longitudinal assessment of respiratory mechanics by oscillometry is feasible, produces reproducible results in patients receiving NIV and provides information on lung condition that may help physicians to evaluate disease progression, titrate and follow response to treatments. Future studies, including more patients with different clinical conditions, should further address this last concept.

Our study has limitations. Firstly, we studied a convenience series of patients and this can result in a selection bias. We included a small number of patients with heterogeneous clinical conditions. However, the heterogeneity and the high BMI of our subjects reflects a real-life hospitalised population in the COVID 19 ward. Moreover, as all patients in our convenience series improved and were discharged to medical wards we have no data to evaluate whether changes in lung mechanics may be predictive of NIV failure or mid-to-long term respiratory outcomes. Future studies should address this point.

In conclusion, our data showed that FOT is well tolerated by hypoxemic COVID-19 ARDS patients receiving NIV, suitable for being included in clincal management of such patients and provides reproducible data. Our data confirm that the COVID-19 ARDS hypoxemia is associated with heterogeneous mechanical lung conditions and longitudinal assessment of respiratory mechanics by oscillometry may constitute an additional tool to improve tailoring of treatments in settings where lung function data is unavailable. This may be especially valuable in the patients with respiratory comorbidities, congestive harth failure [33] or other conditions affecting lung mechanics. Further studies should be addressed to assess the clinical value of oscillometry in patients with ARDS due to COVID-19 receiving NIV.
